# A magnetohydrodynamic mechanism for the formation of solar polar vortices

**DOI:** 10.1073/pnas.2415157121

**Published:** 2024-11-11

**Authors:** Mausumi Dikpati, Breno Raphaldini, Scott W. McIntosh, Marianna B. Korsos, Gustavo A. Guerrero, Peter A. Gilman

**Affiliations:** ^a^High altitude Observatory, NSF-National Center for Atmospheric Research, Boulder, CO 80301; ^b^Lynker Space, Boulder, CO 80301; ^c^Department of Automatic Control and Systems Engineering, The University of Sheffield, Sheffield S1 3JD, United Kingdom; ^d^Physics Department, Universidade Federal de Minas Gerais, Belo Horizonte, MG 31270-901, Brazil

**Keywords:** polar vortices, geophysical fluid dynamics, magnetohydrodynamics, solar flows, solar cycle

## Abstract

Polar vortices are ubiquitous features of planetary atmospheric flows, from the Earth-like rocky planets to Jupiter- and Saturn-like gas giant planets. Very little is known about their existence or dynamics on the Sun. What should be expected near the Sun’s pole for the upcoming solar multi-viewpoint and polar missions? Here, we report the magnetohydrodynamic (MHD) nonlinear simulations for the formation and evolution of solar polar vortices using a near-surface MHD shallow-water model. Our findings indicate that the rush to the poles, the migration of magnetic fields toward the pole following the Sun’s magnetic cycle, can positively contribute to the formation of polar vortices. The mechanism proposed here for the formation of polar vortices involves the role of magnetic fields and may be relevant to any star with a magnetic cycle. The Sun’s polar vortices resulting from this mechanism are predominantly MHD, consisting of a tight pair of cyclonic and anticyclonic swirls. This mechanism is likely to operate during all solar cycle phases except the peak, when the polar field reverses. Polar vortices can impact dynamical evolution of global flows and polar fields, which seed the next activity cycle, hence better knowledge of physics of polar regions may lead to improved solar cycle and space weather forecasts.

## Significance

Polar vortices are omnipresent in planetary atmospheres, however, not much is known about their existence and characteristics in the Sun due to the present lack of direct observations at the poles. Unlike planetary atmospheres, the subsurface layers of the Sun are highly influenced by the presence of magnetic fields. Here, we show that solar cycle magnetic fields provide a plausible mechanism for the formation of polar vortices in the Sun. Observing polar regions of the Sun could provide important clues for understanding the origin of solar magnetism as well as its cyclic behavior. Our results would provide observational targets for multi-viewpoint polar missions, which can give a glimpse of flows and magnetic fields in polar regions.

Polar vortices have been observed in several planets, both rocky and Gas Giants. On Earth, the polar vortices play an important role in weather and have rich dynamics. In periods when they are strong and stable, they keep cold air confined to the poles; on the other hand, when they weaken, they may become more wavy, eventually advecting cold air from the poles to mid-latitudes causing cold spells (1). In Jupiter, the Juno mission observed both north and south poles and recently revealed a rich structure and dynamics, with the north-pole vortex surrounded by eight circumpolar cyclones/anticyclones, while the south-pole is surrounded by five circumpolar vortices (2). In Saturn, the Cassini spacecraft observed both poles, revealing a remarkable hexagonal shape that is evident in the northern hemisphere, while the southern vortex assumes a more circular shape (3). Polar vortices were also observed in Mars, Venus, Uranus, Neptune, and Titan (4).

Although the aforementioned planets possess very different sizes, rotation rates, and atmospheric composition, polar vortices are a common feature of their atmospheric dynamics. In the Sun, no direct observation of polar vortices has been performed due to the lack of multi-viewpoint and/or polar missions. The only mission in the next decade targeting the Sun’s poles is the Solar Orbiter (5). Therefore, the important questions remain from both observational and theoretical points of view: Does the Sun have polar vortices? If so, what might they look like? Do they evolve with the solar cycle? Previous helioseismic observations provide evidence about the presence of dominant m = 1 mode at ~75°, although they lack polar observation (6, 7).

While a definitive answer can only be confirmed by a mission that would directly observe the Sun’s poles, we can anticipate answers by using numerical simulations to infer the characteristics of polar vortices likely existent in the Sun. There are important differences between the dynamics of the flows in the Sun and planets. The most important is that the Sun contains strong magnetic fields, being composed of ionized plasma. Therefore, it is expected that the magnetic fields will, in some form, affect the existence/nonexistence, morphology, and evolution of polar vortices. Both large- and small-scale magnetic fields in the Sun are very dynamic and follow the well-known ~11-y sunspot cycle, while the polar fields (dominated by the dipole component) complete a cycle every ~22 y. Shorter periods’ magnetic variability, such as Rieger-Type (8), quasi-annual (9), and quasi-biennial (10) periodicities also impact significantly the overall strength and morphology of the solar magnetic fields and influence the “seasons” of space weather (11).

The presence of a polar vortex in the Sun would impact the understanding of the solar cycle. Different morphologies of the vortices would imply differences in the poleward advection of magnetic fields influencing the evolution of the magnetic cycle. Furthermore, due to the prevailing concept in the community that the polar field is the precursor to the upcoming cycles, monitoring its evolution is an essential part of long-term space weather forecasting (12).

Although high-latitude m = 1 modes have been observed (13), it is not known whether a solar polar vortex with tight swirling flows would reach deep into the convection zone, or be a near surface phenomenon. Recent studies (14) have found evidence of polar latitude swirls of longitudinal wave number m = 1 in their linear hydrodynamic eigensystem for solar inertial oscillations in the whole convection zone going up to a rigid top, placed at 0.985 $R_{⊙}$, in their model. Here, $R_{⊙}$ represents one solar radius. It focuses on deep inertial and convective modes with deep structures and long time scales. We focus on mechanisms that have not yet been investigated, namely the global magnetohydrodynamic (MHD) effects in a thin shell located at the surface layers, extending from 0.97 $R_{⊙}$ −1 $R_{⊙}$, where there is evidence for quasi-Two-dimensional (2D) (latitude-longitude) Rossby wave modes (15).

Planetary vortices have been simulated using hydrodynamic shallow-water models (16–18). Jupiter’s atmosphere, though thin, has underlying layers that are also fluid, extending for several scale heights and contains small-scale convection. Shallow-water models have the advantage of “filtering out” small-scale nonhydrostatic buoyant convection, to focus on more global quasihorizontal dynamics. For the Sun, we are at a much earlier stage in the development of models and also in finding evidence of such polar vortices. Hence, it is appropriate to consider a wider range of physical scenarios that could lead to solar polar vortices.

We use a MHD shallow-water model to simulate the evolution and morphology of the solar polar vortices under different scenarios of surface magnetic field configurations. We are interested in the uppermost optically opaque part of the Sun where observations will eventually be available, the supergranule layer. Simulations are performed with varying strengths of magnetic fields to mimic the conditions for different stages of the cycle: in the absence of a large-scale polar magnetic field, compatible with a peak phase scenario, with a strong field of 16 G, representative of a strong sunspot cycle’s minimum scenario, and an intermediate case with a field of 5 G that could represent a typical descending cycle scenario.

## Results

We solve the MHD shallow-water equations in a thin Three-dimensional (3D) spherical shell at the surface with an inner radius of 0.97 $R_{⊙}$ and with a thickness of 0.03 $R_{⊙}$. A background mean flow is set to match the Sun’s surface differential rotation (i.e., $\Omega \left(\phi \right)=[451.1-63.\sin^{2}\phi$ $-66.7\sin^{4}\phi -432.]$, ϕ is the latitude). We select the radius of the shell and the inverse of core-rotation rate as unit length and unit time, respectively, in nondimensional units. Thus, 100 units of nondimensional time approximately corresponds to 1-y dimensional time (details are provided in the *Methods* section).

**Fig. 1.**
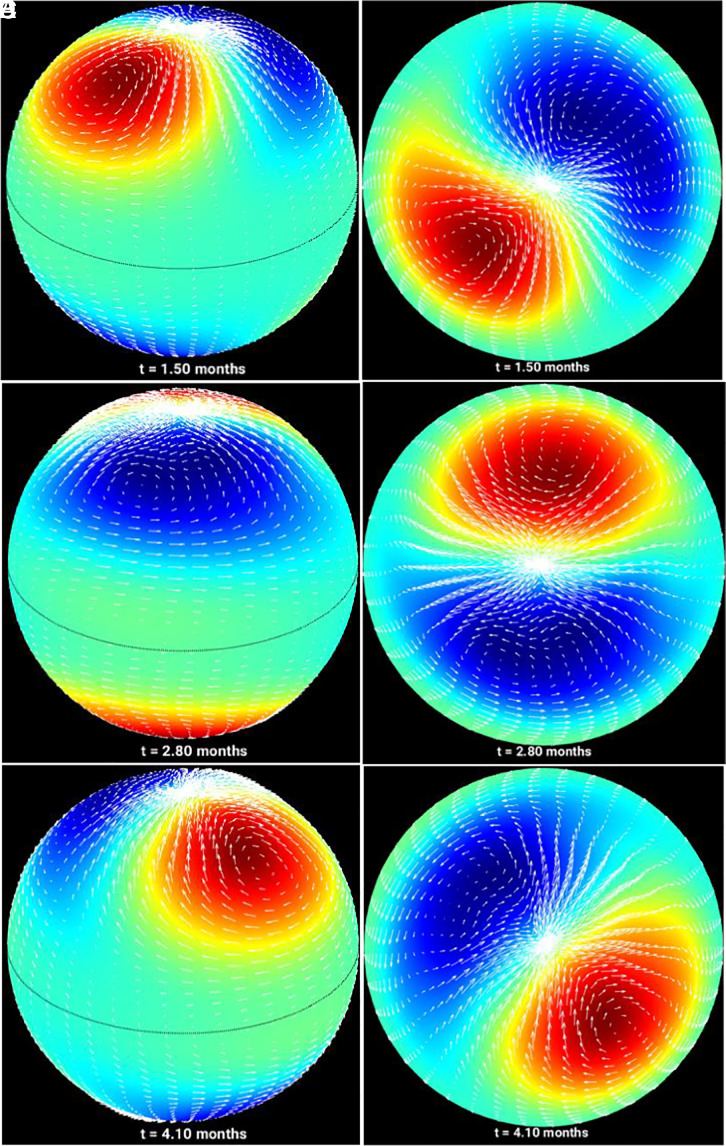
Simulations of global vortices generated hydrodynamically: 30° inclined view (*Left*) and polar view of the spherical shell (*Right*) for the hydrodynamic simulations showing that the perturbations remain in the form of broad large-scale vortices that propagate retrograde similar to hydrodynamic Rossby waves. Arrow vectors represent the vortical flows; color-maps show layer thickness variation in terms of bulging (red) and depression (blue) [or pressure departures from unperturbed pressure—high-pressure (red) and low-pressure (blue)]. The snapshots are taken at the times 1.50 months (panels *A* and *D*), 2.80 months (panels *B* and *E*) and 4.10 months (panels *C* and *F*) during the evolution vortices.

First, we describe the outcome of the hydrodynamic case. In each of the simulations, the initial conditions are set according to the most unstable linear eigenmodes from the (M)HD shallow-water model with the solar differential rotation profile. Initialization by the most unstable mode of the system for starting the nonlinear evolution is a usual practice and has been heavily used in oceanic and atmospheric shallow-water model simulations (19). In this particular (hydrodynamic) case, m = 1 represents the most unstable mode; we accordingly start the nonlinear evolution of the system starting with initializing the system with an m = 1 mode. Fig. 1 portrays the evolution of the corresponding vortices throughout a 20-mo integration. The solutions in this case are characterized by a pair of broad vortices extending from 50° latitude up to the poles. Such a broad cyclone/anticyclone has not been identified as a polar vortex in general. Thus, the hydrodynamic simulations cannot really produce tight vortices clustered around the poles from mid-latitude perturbations; instead, the large-scale vortices remain broad throughout the entire simulation span and can be understood in terms of classical hydrodynamic Rossby waves (15).

The inclusion of magnetic fields, however, greatly modifies the dynamics of waves in a shallow-water model (20–24). Linear eigenmodes from MHD instability of high-latitude bands provide important guidance suggesting the growth of eigenmodes with substantial energy at high relative to the low latitudes and their nonlinear dynamics must be considered in order to understand likely morphologies of solar polar vortices. Numerical experiments are set to represent the phenomenon called “rush to the poles” (25–30), that consists of migration of polar magnetic fields to higher latitudes as solar cycles progress to their minima phases after the peaks, strengthening the polar fields until the solar minima. In order to represent the polar rush we include a horizontal magnetic field band in the zonal direction, initially at 60° latitude, and moving poleward. Two MHD scenarios are investigated to understand the impact of the magnetic field evolution on the formation of the polar vortices, namely a weak magnetic band of 5 G and a relatively stronger band of 16 G migrating toward the pole with polar rush.

**Fig. 2.**
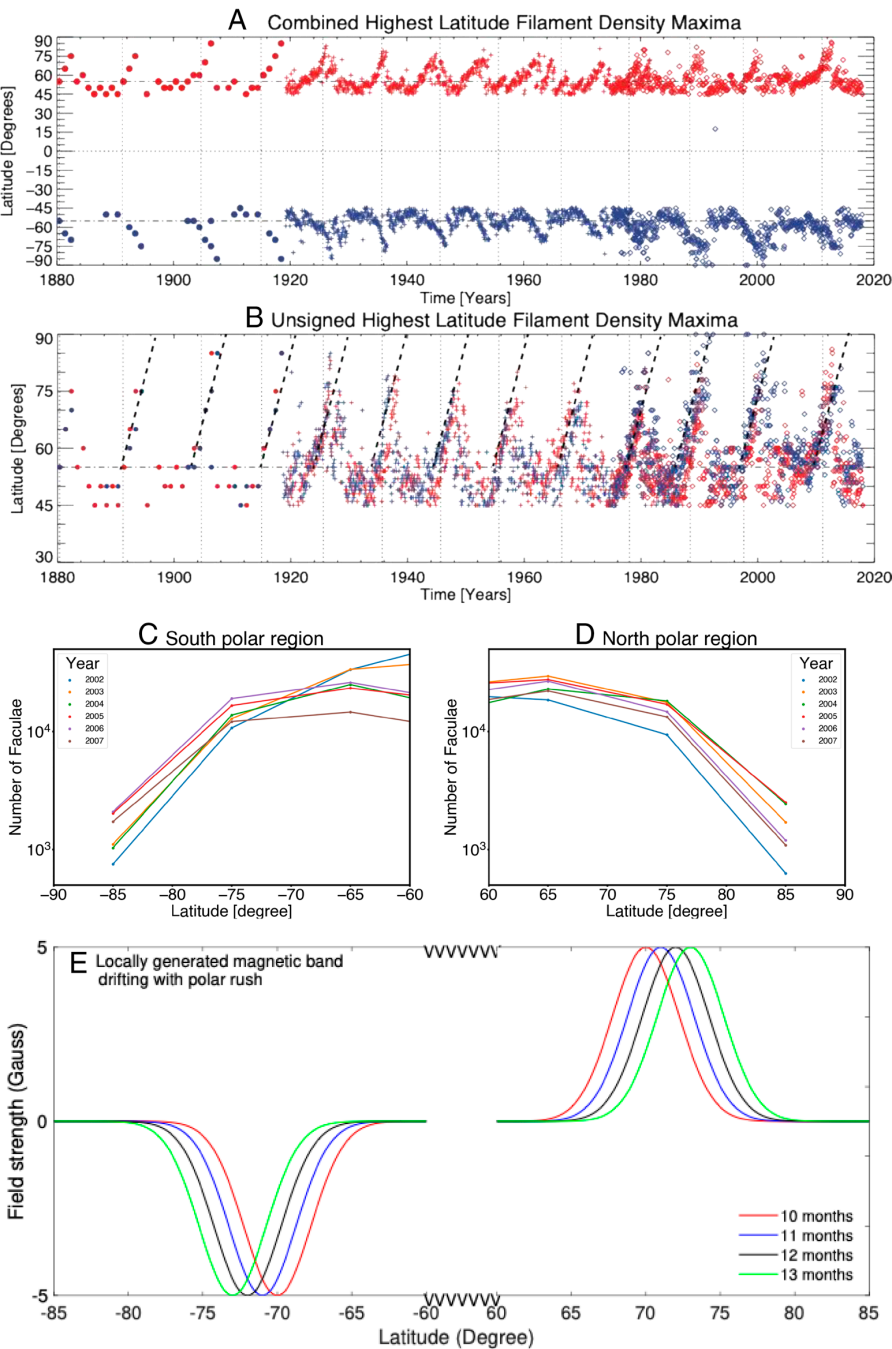
Observational proxies of polar fields, and estimated accompanying toroidal magnetic fields: (*A* and *B*) Solar filament evolution from 1880 to 2017, recreated from data used in ref. 25. Panel *A* illustrates the density of filaments (red—north; blue—south) and panel B the latitude of the highest latitude filaments (hemispheric high-latitude filaments are overlaid). Slanted black-dashed lines in panel *B* denote their speed of the rush to the poles, approximately 1° per month. Reference to ±55° latitude is drawn using a dot-dashed horizontal line, the equator using a dashed horizontal line, and the locations of the Hale Cycle “termination” events as vertical dashed lines. Panels *C* and *D* display the number of emerging faculae as a function of latitude from 60 to 90°. The counted number of faculae has been plotted on a log scale in the north (*C*) and south (*D*) during the declining phase of cycle 23. Each year’s data are represented by a different color, highlighted in the legend. (*E*) displays banded toroidal magnetic fields locally generated from polar fields that drift poleward with the rush to the poles (*A* and *B*).

Fig. 2 *A* and *B* depicts the rush to the poles of the filaments. We overlay in Fig. 2*B* the northern (red) and southern (blue) hemispheric high-latitude filaments in the record to permit a superposed epoch analysis (26). Slanted black-dashed lines in Fig. 2*B* denote the average drift-speed of filaments, approximately 1°/mo. Filaments’ migration is a proxy for the migration of polar fields to the poles, starting from the latitudes ~55°, as the cycle progresses into its declining phase after the peak (26). We can see that the latitude-range of polar filaments at any given time is narrow, implying a narrow latitude-structure of polar fields. The polar rush phenomena is not yet well-understood theoretically. Some suggestions combine diffusion of magnetic flux from decaying active regions and the transport by photospheric flows (31, 32). Notwithstanding occasional reverse flow cells, likely present in the global meridional circulation, the polar rush plays an important role in the dynamics there.

Fig. 2 *C* and *D* displays the latitude-structure of polar fields, from the proxy of faculae counts, in the north (C) and south (D). The Solar and Heliospheric Observatory Michelson Doppler Imager (SOHO/MDI)–Debrecen Faculae Data Catalog (Helioseismic and Magnetic Imager Debrecen faculae Data catalogue (HMDD)) was employed from 1997 to 2008 (32). The differential rotation near the surface of the Sun will shear the polar(poloidal) field, generating a toroidal field component through the well-known “Ω-effect” (33). In contrast to Fig. 2 *A* and *B*, since Fig. 2 *C* and *D* are annual averages, the faculae count profiles are much broader in latitude. There exist various observations with shorter term averaging that indicate polar fields have a somewhat narrower latitudinal width (34–36) than Fig. 2 *C* and *D* but wider than Fig. 2 *A* and *B*.

Weak toroidal fields of latitudinal width intermediate between that in Fig. 2 *A*–*D*, drifting poleward following the global dipole field, have indeed been observed (37). We simulate the MHD shallow-water dynamics of such banded toroidal fields of magnitudes of a few Gauss, produced by this shearing process in a few weeks. As depicted in Fig. 2 *A* and *B*, the polar rush phenomena is highly dependent on the strength of the cycle (and hence on the zonally averaged magnetic field strength). The representation of this phenomenon on the formation of polar vortices is investigated by considering two scenarios, 5 G and 16 G.

For the integration with the magnetic field strength of 5 G migrating poleward, the conditions mimic a situation of the descending phase of a typical solar cycle. In this case, the initial condition is chosen to be a m = 4 mode, since it constitutes the most unstable mode with the solar differential rotation and this toroidal field configuration. The morphology of the toroidal magnetic field in latitude consists of a narrow band with a Gaussian decay away from the peak magnetic field as depicted in Fig. 2*E*. Throughout the 20-mo integration, as the magnetic field band moves poleward, the flow pattern undergoes nonlinear interactions and the dominant pattern changes from m = 4 to a superposition of m = 1 and m = 2 modes, with the m = 1 being the dominant one, occasionally exchanging energy with a weak m = 2 mode. Specifically, the magnetic band is moved by ~0.0001° after every integration time step (i.e., after every 0.001 time-unit, which corresponds to ~5 min). This drifting is performed by extracting the m = 0 components of both the zonal and meridional magnetic fields. Although at time t = 0, the m = 0 component is purely zonal, the corresponding m = 0 meridional component develops through nonlinear evolution of the system. Hence, by performing this procedure, we implement the latitude drift of the axisymmetric fields following polar rush.

**Fig. 3.**
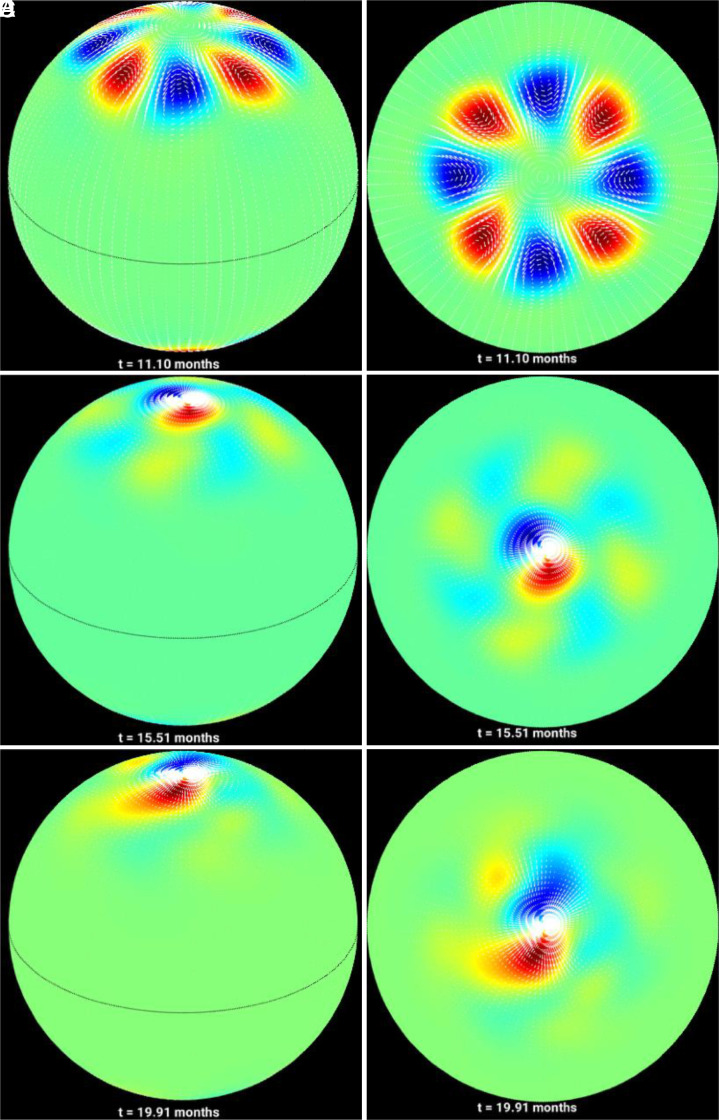
Simulations of magnetohydrodynamically governed polar vortices for typical solar cycles: Same as in Fig. 1, but for the weak field MHD simulations showing that the initial mid-latitude perturbations tend to drift and cluster around the poles following the rush to the poles. An initial m = 4 perturbation yields a final configuration consisting of a quasistable pattern with a dominant m = 1 mode (a pair of vortices) along with occasional appearances weak m = 2 and also sometimes m = 3 modes (Movie S1). The snapshots are taken at the times 11.10 months (panels *A* and *D*), 15.51 months (panels *B* and *E*) and 19.91 months (panels *C* and *F*) during the evolution vortices

The Fig. 3 shows the evolution of the flow pattern in arrow vectors throughout the 20-mo integration and highlights the concentration of energy near the poles as the rush to the poles process advances, as well as the establishment of the m = 1/m = 2 patterns toward the solar minima configuration of the magnetic field. The physics behind this concentration of energy near the poles lies in that Rossby wave energy is trapped near the magnetic field bands, i.e., the magnetic field band constitutes a waveguide for magnetized Rossby waves (20).

As the magnetic band moves toward the pole, waves’ energy follow the magnetic band’s drift. The vortical flows more or less maintain the magnetostrophic balance, except at the times of appearance of inertia-gravity waves.

**Fig. 4.**
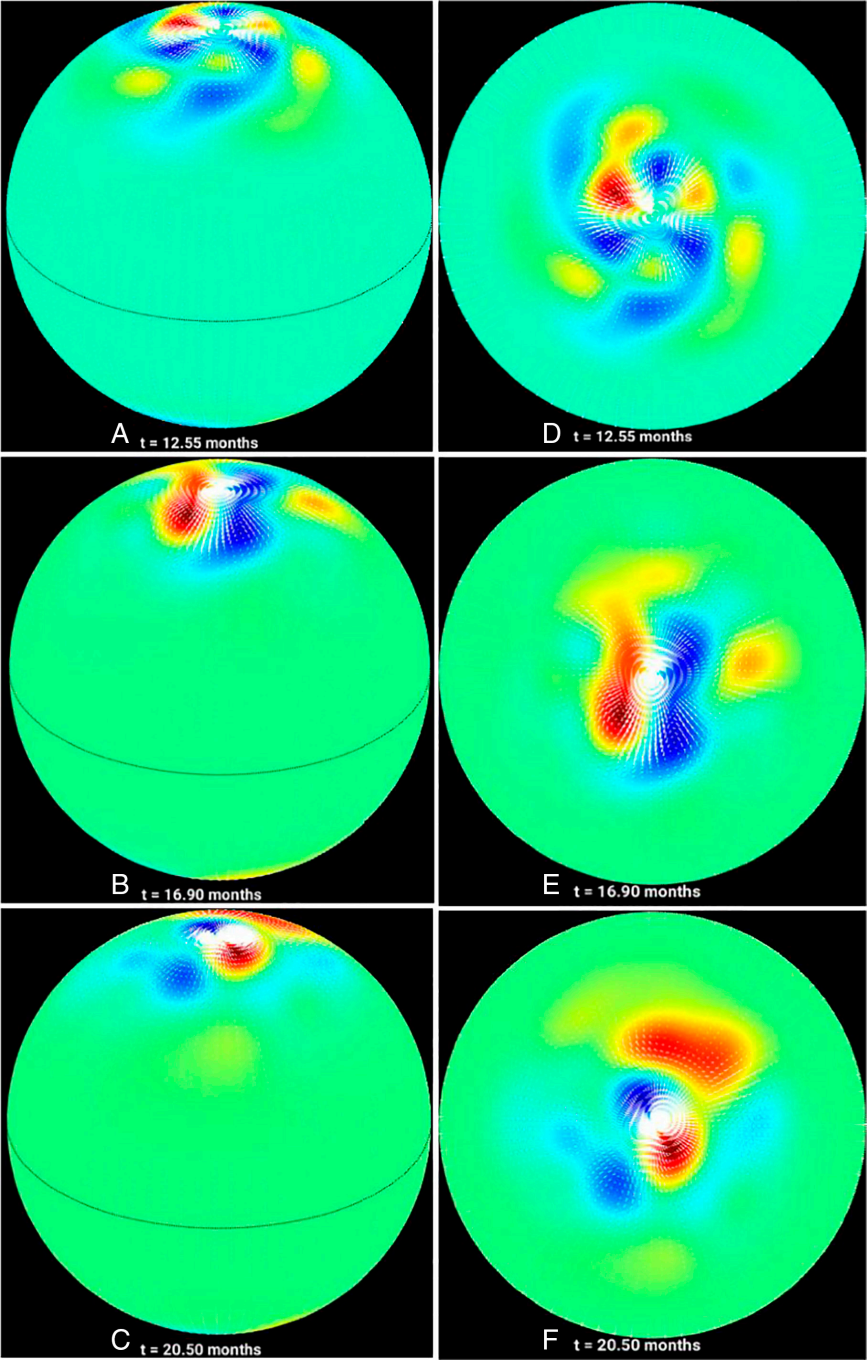
Simulations of magnetohydrodynamically governed polar vortices for strong cycles: 30° inclined view (*Left*) and polar view (*Right*) of the poles for the strong field MHD simulations showing that the initial mid-latitude perturbations tend to drift and cluster around the poles following the rush to the poles. An evolved configuration consisting of a dominant m = 1 pattern (a pair of vortices) is obtained from an initial m = 3 perturbation. The snapshots are taken at the times 12.55 months (panels *A* and *D*), 16.90 months (panels *B* and *E*) and 20.50 months (panels *C* and *F*) during the evolution vortices

The strong field case, with a 16 G magnetic field band, is displayed in Fig. 4. The initial condition consists of a m = 3 mode. Similar to the weak field case, the vortices become progressively clustered near the poles. Here, the flow pattern develops two circumpolar rings of nonlinearly interacting vortices, each consisting of three cyclonic and three anticyclonic swirls, persisting for a significant timespan. As the simulation further progresses with the magnetic band drifting closer to the pole, eventually a dominant m = 1 mode forms like the weak field case, but with a significant m = 2 component arising from nonlinear mode–mode interaction.

**Fig. 5.**
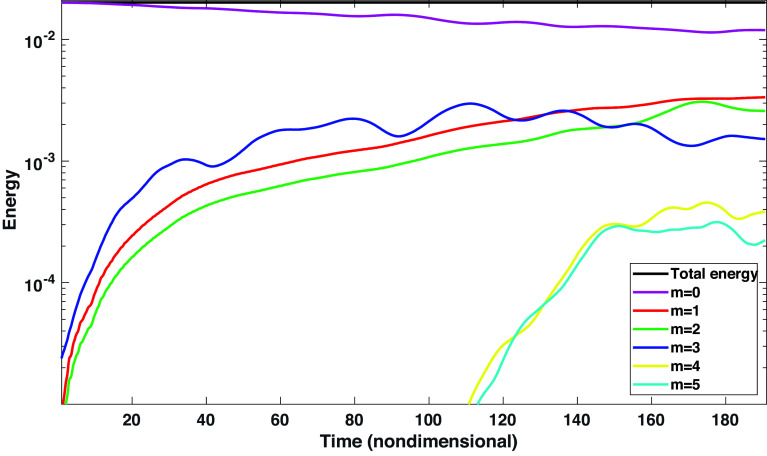
Evolution of energies among various longitudinal modes: Evolution of total energy (primarily dominated by kinetic energy) associated with each mode (m = 0 to 6) has been displayed. 100 units of nondimensional time corresponds to ~1 y. While modes interact nonlinearly among themselves, near the end of simulation, when the magnetic fields have reached a very high-latitude (~85°) with polar rush, m = 1 modes dominate over m = 2 and 3.

For this last case (with 16 G field strength), we compute the total energy of the system following (20). In these experiments, the energy is primarily dominated by the kinetic energy, because potential and magnetic energies are a few orders of magnitude smaller. We display in Fig. 5 the total energy evolution associated with each wavenumber up to 5 (m = 0 to 5). Nonlinear exchanges of energies among these modes can be seen in Fig. 5. The energy associated with even higher modes (m > 5) is significantly smaller; the total energy is conserved to a great accuracy. Note that the amplitudes of the corresponding modes can be obtained by approximately taking the square-root of energies associated with the respective modes.

## Discussions

Polar vortices are observed in virtually all planets and satellites that possess an atmosphere (4). Stars, and in particular, the Sun, have the same ingredients present in planetary atmospheres, namely, a fluid in presence of Coriolis force, suggesting that they could also possess polar vortices. However, the presence of strong magnetic fields is known to significantly alter the dynamics of rotating fluids (plasmas). In particular, near the surface of the Sun, magnetic fields at higher latitudes are characterized by the rush to the poles phenomena.

Here, we report results from simulations where we investigate the role of magnetic fields in the formation of polar vortices in the Sun from initial mid-latitude perturbations. Simulations are performed in order to represent three different scenarios i) a hydrodynamic case without the presence of magnetic fields, ii) a typical cycle’s magnetic field case where a 5 G band drifts poleward with the polar rush, and iii) a strong field case with an evolving toroidal field with the same configuration as in the weak field case, but with magnetic field strength of 16 G.

Our results demonstrate that in the hydrodynamic case i), tight polar vortices, like that in Jupiter’s poles, are not formed from an initial mid-latitude perturbation; instead, broad large-scale cyclonic/anticyclonic flows nonlinearly evolve in a quasistable fashion throughout the several months’ integration period; these retrograde-propagating vortical patterns can be explained as a dominant m = 1 hydrodynamic Rossby/inertia-gravity mode. In a typical cycle, i.e., for the magnetic field case ii), the poleward drift of magnetic field is accompanied by a progressive clustering of the vortices around the poles, starting from a m = 4 initial perturbation and resulting in a quasistable state characterized by a dominant m = 1 mode. Finally, in the strong field case iii) the initial m = 3 perturbation also evolves to a configuration of clustering of the vortices near the poles, with an eventually evolved state, consisting of mostly m = 1 mode.

When comparing our results with the variability of the magnetic fields throughout different solar cycles, in particular, the variability of the polar rush phenomena, we may associate the different configurations in the simulations with weak or strong cycles. Our main conclusions are that the formation of polar vortices on the solar surface from mid-latitude perturbation are strongly influenced by the presence of magnetic fields and that the final configuration of the polar vortex in the simulations presented here (compatible with all solar cycle phases, except the peak) crucially depends on the strength of the drifting background magnetic fields. Nevertheless, it remains to be explored the relevance of three-dimensional dynamics on the formation of vortical structures and the role of the magnetic field on their evolution.

Helioseismology has previously inferred the presence of inertial modes with dominant wavenumber m = 1 at high latitudes of about 75° (6). The frequency for the m = 1 we found here (of −86.8 nHz) is comparable with the linear speeds in previous studies (6). Recent theoretical models aimed at explaining these high-latitude inertial modes do not find Jupiter-like polar vortices and did not include polar magnetic fields (38). In contrast, our work indicates that the presence of polar magnetic fields favors the formation of tight vortices at the Sun’s poles.

We note that different mechanisms may contribute to the formation of planetary polar vortices, but often, they rely on the principle of conservation of vorticity allied with some form of meridional transport due to differential latitudinal atmospheric heating (4, 11, 22). The Jupiter simulations were initialized with finite amplitude vortices of specified potential vorticity, sometimes with one or more added small “seed” vortices, and simulates how the initial vortices rearrange via nonlinear dynamics into stable, regular patterns of vortices that circle the pole, with total potential vorticity conserved for all the vortices present. The resulting stable configurations strongly resemble the observed ones. By contrast, the mechanism proposed here for the formation of polar vortices on the Sun is governed by weak magnetic fields. In the MHD case, there are electromagnetic body forces which can impart vorticity to the fluid by applying torques to fluid elements, causing both the local and the total potential vorticity of the fluid to change. These torques are periodic in longitude; so, adjacent vortices are driven by corresponding oppositely directed torques about the local radial axis.

However, in both cases, it is the Rossby waves’ dynamics that plays the central common role—in planets, they may be generated by baroclinic instability by differential heating, whereas in the Sun they are magnetically modified Rossby waves, generated by instability of differential rotation. The energy comes primarily from the kinetic energy of differential rotation and magnetic energy to drive the nonlinear evolution of vortices through exchanges of energy among these energy reservoirs and that of Rossby waves (39). In the hydrodynamic case, the cyclonic/anticyclonic flows are wider, because the energy source is primarily kinetic, coming from the broad solar differential rotation. In the MHD case, relatively more complicated nonlinear exchanges of energy among magnetic and kinetic energy reservoirs (39) as well as Rossby mode–mode interactions occur (40), resulting into tight swirls of cyclonic/anticyclonic vortices, evolving between dominant m = 1 and weak m = 2 patterns.

## Concluding Remarks

Previously proposed mechanisms for the formation of high-latitude vortices in the Sun are based on linear hydrodynamic eigenmodes in a model of the bulk of the convection zone with rigid top and bottom, taken respectively at 0.7 $R_{⊙}$ and 0.985 $R_{⊙}$ (14). Near the surface, however, the dynamics is affected by magnetic fields. Currently, it’s not known whether or how the dynamics of this thin subsurface layer as well as the Sun’s magnetic cycle influence the formation of vortices in the polar region of the Sun.

Here, we used a nonlinear MHD shallow-water model to show that the magnetic cycle, and in particular, the rush-to-the-poles phenomena can favor the formation of polar vortices from mid-latitude perturbations. Our major finding is that as the sunspot cycle progresses, a ring of vortices will form near the latitude of highest polar fields, associated with the rush to the poles, and then, the ring of vortices will shrink toward the poles, shedding vortices as it moves, leading ultimately to a single pair of vortices very near the poles, which disappear during solar maximum, when the polar field is zero. Therefore, polar vortices are most likely to be observed during all phases of the solar cycle except perhaps the peak phase.

The mechanism proposed here for the formation of polar vortices fundamentally relies on the nonlinear interplay between magnetic fields and flows. Exploring other plausible physical mechanisms, such as baroclinic instabilities (41), and the effects of deeper layers of the Sun, such as the bulk of the convection zone (14), is beyond the scope of our present model. As such, our mechanism presented here and the previously suggested ones are by no means exclusive, since they concern different regions of the Sun, and if combined, they could reinforce the formation of polar vortices. Only comparison with observations from multi-viewpoint solar polar missions could provide further insights into the relative contributions of each of these mechanisms.

The existence of the solar polar vortices would impact the polar latitudes’ differential rotation by wave-mean flow interactions, hence spinning-up and spinning-down the poles as the high-latitude vortices lose stability. This could affect the global meridional circulation also. It is a widely accepted view in the community that the polar fields are precursors of the strength of the upcoming cycle (12); furthermore, vortical flows near the photosphere could amplify the polar magnetic fields (42) before it becomes the seed to the next cycles. However, without better knowledge of the properties and dynamics of polar region’s flows and fields the physical foundation of this view may not be justified. How does the polar vortex actively interact and participate in the polar field evolution? Obviously, the answers to such questions lie in the multi-viewpoint observations of high-latitude regions, from about 60° to the poles. A promising direction for incorporating as many observations as possible into numerical models has begun, via data assimilation, machine learning, deep learning, and AI (43).

While the Solar Orbiter will give the first glimpse of the high-latitude magnetic field and flow in 2025, it does not have sufficient viewing of the polar regions. The Polarimetric and Helioseismic Imager on Solar Orbiter could improve the measurements from a 30° out-of-ecliptic orbit but does not provide the temporal coverage required to fully trace the evolution of the magnetic concentrations through the cycle. This highlights the need for future multi-viewpoint polar missions that are able to observe the poles during times other than solar maximum.

## Methods

### Model and Equations.

The main starting point for our simulation is a shallow-water model, which is a quasi-three-dimensional thin shell system of fluid, the core foundation of which is that the horizontal scale and motions are very large compared to the vertical extent and motions in it. This renders the pressure perturbations to be hydrostatic. Such models have been widely used in studying global dynamics of planetary atmospheres and oceanic systems (see e.g., ref. 44, for details). Such a system has been employed for studying certain global dynamics of the Sun over the past two decades with the generalization from a global hydrodynamic shallow-water system to a MHD one, because the Sun contains very strong magnetic fields, which govern the global solar dynamics. Complete foundation for a global MHD shallow-water system and the full set of equations are presented in vector invariant form in ref. 45 (see e.g., their equations 3, 5, 6, and 8) which have subsequently been employed by various authors in rectangular (46, 47) as well as in spherical systems (21, 23, 24, 48).

Note that hydrostatic shallow-water models, which have been used to simulate Jupiter’s polar vortices (16), filter out small-scale buoyant convection, in order to focus on more global-scale nearly horizontal dynamics. The supergranulation layer is a thin layer near the solar surface, in which the horizontal extent and motions are much larger than that in the radial direction (49). Thus, the dynamics occurring there can satisfy such foundation criteria of a shallow-water model. Considering a thin shell extending from 0.97 $R_{⊙}$ to 1 $R_{⊙}$, a hydrodynamic shallow-water model has recently been employed to simulate classical Rossby waves (15), which were detected there by helioseismology (50). Here, we generalize that model to include MHD for simulating the polar region’s MHD of the global flows, primarily focusing on whether cyclonic/anticyclonic tight swirls can form.

By denoting the latitude (*ϕ*) and longitude (*λ*) components of velocities and those components of magnetic fields, respectively as *u*, *v*, *a*, *b*, and the shell thickness as 1+, we present the full set nonlinear MHD shallow-water equations in the nondimensional form in the rotating frame, rotating with the core-rotation rate ($\omega_{c}$):

[1] $$\begin{array}{c} \frac{\partial u}{\partial t}=\frac{v}{cos\phi }\left[\frac{\partial v}{\partial \lambda }-\frac{\partial }{\partial \phi }(u\;cos\phi )\right]-\frac{1}{cos\phi }\frac{\partial }{\partial \lambda }\left(\frac{u^{2}+v^{2}}{2}\right)-G\frac{1}{cos\phi }\frac{\partial h}{\partial \lambda }+2\;\omega_{c}\;v\;sin\phi \\ \;-\frac{b}{cos\phi }\left[\frac{\partial b}{\partial \lambda }-\frac{\partial }{\partial \phi }(a\;cos\phi )\right]+\frac{1}{cos\phi }\frac{\partial }{\partial \lambda }\left(\frac{a^{2}+b^{2}}{2}\right), \end{array}$$

[2] $$\begin{array}{c} \frac{\partial v}{\partial t}=-\frac{u}{cos\phi }\left[\frac{\partial v}{\partial \lambda }-\frac{\partial }{\partial \phi }(u\;cos\phi )\right]-\frac{\partial }{\partial \phi }\left(\frac{u^{2}+v^{2}}{2}\right)-G\frac{\partial h}{\partial \phi }-2\;\omega_{c}\;u\;sin\phi \\ \;+\frac{a}{cos\phi }\left[\frac{\partial b}{\partial \lambda }-\frac{\partial }{\partial \phi }(a\;cos\phi )\right]+\frac{\partial }{\partial \phi }\left(\frac{a^{2}+b^{2}}{2}\right), \end{array}$$

[3] $$\frac{\partial (1+h)}{\partial t}=-\frac{1}{cos\phi }\left[\frac{\partial }{\partial \lambda }\left(\left(1+h\right)u\right)+\frac{\partial }{\partial \phi }\left(\left(1+h\right)v\;cos\phi \right)\right],$$

[4] $$\frac{\partial a}{\partial t}=\frac{\partial }{\partial \phi }\left(ub-va\right)+\frac{a}{cos\phi }\left[\frac{\partial u}{\partial \lambda }+\frac{\partial }{\partial \phi }\left(v\;cos\phi \right)\right]-\frac{u}{cos\phi }\left[\frac{\partial a}{\partial \lambda }+\frac{\partial }{\partial \phi }\left(b\;cos\phi \right)\right],$$

[5] $$\frac{\partial b}{\partial t}=-\frac{1}{cos\phi }\frac{\partial }{\partial \lambda }\left(ub-va\right)+\frac{b}{cos\phi }\left[\frac{\partial u}{\partial \lambda }+\frac{\partial }{\partial \phi }\left(v\;cos\phi \right)\right]-\frac{v}{cos\phi }\left[\frac{\partial a}{\partial \lambda }+\frac{\partial }{\partial \phi }\left(b\;cos\phi \right)\right],$$

[6] $$\frac{\partial }{\partial \lambda }\left(\left(1+h\right)a\right)+\frac{\partial }{\partial \phi }\left(\left(1+h\right)b\;cos\phi \right)=0.$$

All variables (*u*, *v*, *a*, *b*, *h*) are functions of latitude (*ϕ*), longitude (*λ*) and time (*t*). Eqs. **1** and **2** comprise the evolution of two horizontal velocity components (*u*, *v*), and (Eq. **3**) provides the evolution of top surface, from which radial component of the velocity can be derived. Two horizontal magnetic field components evolve following Eqs. **4** and **5**, and the modified zero-divergence of magnetic field condition is given by Eq. **6**, which can be used to derive the vertical magnetic fields. *G* is a nondimensional parameter which represents the “effective gravity” of the model. Bringing the analogy of Jupiter’s polar vortex model (16), we recall that those models have been characterized by a nondimensional number, the so-called Burger number, $B_{u}=\frac{g_{j}H_{j}}{(2\Omega_{j}sin\varphi_{j}L{)}^{2}}$, in which *L* is a horizontal length scale, characteristic of horizontal flow in the spherical shell, *g_j_*, *H_j_*, $\Omega_{j}$, $\varphi_{j}$, respectively, represent Jupiter’s gravity at the shell location at its surface, shell thickness of the shallow-water model employed, Jupiter’s rotation rate, and latitude. The nondimensional parameter in our case is *G*, $\left(G=\frac{gH}{(r_{0}\omega_{c}{)}^{2}}\right)$, which is related to the actual gravity (*g*) at the radius of the bottom boundary, shell thickness (*H*), inner radius of the shell $r_{0}=0.97\;R_{⊙}$ and the core-rotation rate ($\omega_{c}$) (48). Note that our *G* parameter is closely related to the Burger number but does not include any flow length scale; this is replaced by an external parameter, the solar radius ($R_{⊙}$). In a hydrostatic shallow-water model, *G* determines how much the upper boundary of the shallow layer is allowed to deform; for *G* > 1, this deformation is small compared to the shell thickness. For *G* < 1, the deformation allowed increases, with declining *G*, to the point the shell thickness vanishes at certain points that becomes unrealistic for Jupiter (16) and also will be for the Sun. Therefore, we focus on values *G* > 1, primarily *G* = 3, very much like the hydrodynamic shallow-water model implemented in the supergranulation layer for simulating Rossby waves via inverse cascade (15). This implies that the motions in this layer are primarily horizontal, i.e., in latitude-longitude, with small radial motions.

We also briefly mention how the length scale and the time scale are selected to nondimensionalize the system. We consider the radius of the inner boundary (0.97 $R_{⊙}$) to be a unit length and the inverse of core-rotation rate a unit time. This means for the core-rotation rate of 432 nHz, $\omega_{c}=2\pi \times 432$, and a unit nondimensional time corresponds to 1/(2π × 432 × 10^−9^) s in dimensional form, which comes out to be ~0.01 y. Thus, 100 nondimensional units of time correspond to 1 y. Taking a typical supergranulation layer mass density, it is easy to compute the nondimensional field strength in this model; a dimensionless magnetic field of one unit corresponds to a field strength of about 100 G. We consider 0, 0.05, and 0.16 units of field strength for our simulation experiments. These correspond to zero, 5 G, and 16 G toroidal field strength at the surface.

### Observations Invoked for Setting Up Simulation Experiments.

Given the description of the model in the previous section, our next task is to describe the simulation experiments we perform here. We consider a solar-like surface differential rotation (Ω) of the form $\Omega \left(\phi \right)=\left[451.1-63.\sin^{2}\phi -66.7\sin^{4}\phi -432.\right]$ nHz, in the frame rotating with the core-rotation rate of 432 nHz; here, *ϕ* represents latitude. To estimate the structure and amplitude of high-latitude toroidal magnetic fields, as well as their poleward drift with rush to the poles, we take help from observational analysis of polar crown filaments data and faculae concentrations data. Fig. 2 *A* and *B* in the main text presents the drift-speed of the polar crown filaments during their rush to the poles (28).

The Fig. 2 *A* and *B* show approximately 1° per month drift-speed of polar crown filaments, a proxy of the polar fields, during their rush to the poles. While the latitudinal width indicates the latitudinal width of the polar field at a particular time during their rush to the poles, Fig. 2 *C* and *D* in the main text (see also fig. 3 of ref. 34) reveals the observed latitudinal structure of the polar fields from different measures, namely the faculae count, which is another proxy for polar fields. From various observations, the polar fields have already been noted to have banded-type structures in latitude. For example, fig. 3 of ref. 34, derived from faculae concentration in latitude, shows polar (poloidal) fields drifting toward the pole in banded form during the declining phase of cycle 20. A similar observational analysis performed here using SOHO/MDI–Debrecen Faculae Data Catalog (HMIDD) reveals the drift of banded polar (poloidal) fields during the declining phase of cycle 23 (see the slanted vertical line in both panels of fig. 3 of ref. 34).

Thus, we can consider the existence of weak toroidal fields, which are the outcome of the shearing of polar (poloidal) fields by the surface radial differential rotation (e.g., $B_{r}\partial \Omega /\partial r\Delta t$)—their amplitudes can easily be estimated to reach in a few weeks about 5 to 16 G. These weak, surface toroidal fields are not expected to produce spots, and/or be amplified by shearing for a much longer time, because their source, i.e., the polar/poloidal fields, is migrating to a new latitude, and also magnetorotational instabilities will stop their growth. But they will be present with a low amplitude along with the polar (poloidal) fields as long as the polar rush and poleward migration of polar fields continue. Perhaps some of the weak toroidal fields at the surface may be diffusively permeating from the bottom. Nonetheless, such weak toroidal fields should exist along with the polar fields. These weak, surface toroidal fields, of the order of global dipole fields, and reversing with the global dipole field has indeed been observed by ref. 37. As discussed in ref. 37, weak surface toroidal fields either may be locally generated from the shearing of polar (poloidal) fields there, or may permeate from the bottom.

The reversal of surface toroidal fields when the global dipole field reverses indicates that these surface toroidal fields must be drifting toward the pole with the dipole poloidal fields. Without loss of generality in our simulation, the latitudinal width of the weak surface toroidal fields can be prescribed as a Gaussian; spatial structure of polar fields derived from observations also support this choice (see e.g., fig. 3 of ref. 34, also see refs. 35 and 36). If the polar (poloidal) fields are the sources for the observed, weak, surface toroidal fields, they should be present always except when the global dipole field is reversing, i.e., during solar maximum. Therefore, in our simulation setup, we consider three cases: i) no toroidal fields (representing the solar cycle’s maxima phase), ii) a weak toroidal field of 5 G peak-amplitude migrating with a speed of 1° per month from 60 to 85° latitudes (representing all phases of a typical solar cycle except its peak phase), and iii) a toroidal field of 16 G (representing the solar minima phase between two strong cycles).

### Numerical Scheme.

Since detailed numerical schemes for solving global nonlinear (M)HD shallow-water equations have been discussed in refs. 20 and 51, we are presenting the algorithm briefly here. Following the pseudospectral implementation given in ref. 52, the scalar variable *h*is decomposed in scalar spherical harmonics and the vector variables, *u*, *v*, *a*, *b* in vector spherical harmonics, to deal with the pole problem. Nonlinear terms in the equations are computed in a pseudospectral implementation. Adams–Bashforth scheme, with a kick-start by fourth-order Runge–Kutta, is implemented for time evolution; semi-implicit dynamics is included following ref. 53 in order to integrate out high-frequency gravity waves. Momentum is checked and balanced in every few thousand steps, in which the model evolves for about a day. As discussed in the literature, in order to take care of aliasing error (Gibb’s phenomenon) in the pseudospectral formalism, a small numerical viscosity is added as a standard technique. Computer-intensive synthesis and analysis steps are run concurrently in multiple parallel threads on modern, many core processors. In a T42 model, to capture sharp variations of solar surface differential rotation and toroidal fields in latitude and longitude, about 0.2° latitudinal and 2.8° longitudinal resolution has been used.

## Supplementary Material

Appendix 01 (PDF)

Movie S1. An MPEG movie Movie-S1.mp4 has been uploaded.

## Data Availability

Observational data for the polar faculae data used in this study are publicly available from the SOHO/MDI–Debrecen Faculae Data Catalog (HMIDD) (http://fenyi.solarobs.epss.hun-ren.hu/en/databases/SDO/) (54), and of polar rush filaments data from the Arcetri Astrophysical Observatory (for the time 1880 to 1929), Meudon Observatory (1919 to 1989), and the Kislovodsk Observatory (1980 to 2018). All simulation data and codes used to produce figures are deposited in GitHub (https://github.com/mausumidikpati/SolarMHDPolarVortex) (55).
