# Supplementary material for: A magnetohydrodynamic mechanism for the formation of solar polar vortices
Source: Proc Natl Acad Sci U S A. 2024 Nov 11;121(47):e2415157121. doi: 10.1073/pnas.2415157121 (PMC11588063; doi:10.1073/pnas.2415157121)
Supplement: Supplementary file 1 — Appendix 01 (PDF) [file pnas.2415157121.sapp.pdf]

Supporting Information:

A Magnetohydrodynamic Mechanism for the Formation of Solar Polar Vortices

Movie S1: An MPEG movie Movie-S1.mp4 has been uploaded**.**

Caption: Evolution of flows with clockwise and anticlockwise swirls, for the case of 5G field strength, is displayed in Movie-S1 in a 30-degree inclined view of the Sun's north pole, from the time 12.75 months to 16 months, namely when the center of the swirls drift from about 72.7 to 76 degrees. Red/orange in color maps indicate bulges (high pressure regions, positive departure from unperturbed pressure) and blue/sky-blue the depression (low pressure regions). The flows are magnetostrophic, i.e the swirls are clockwise in highs (red/orange) and anticlockwise in lows (blue/sky-blue) in northern hemisphere due to primarily geostrophic balance, with occasional departures due to magnetostrophy. During this evolution, multiple swirling flows eventually turn into two tight polar vortices, centered around approximately 75-degrees. while evolving from approximately from 72.7 to 76 degrees, after which the tight vortices sustain until they reach much closer to the pole (as shown in the bottom panel of Fig. 3).
